# Activation of USP30 Disrupts Endothelial Cell Function and Aggravates Acute Lung Injury Through Regulating the S‐Adenosylmethionine Cycle

**DOI:** 10.1002/advs.202512807

**Published:** 2025-10-17

**Authors:** Baoyinna Baoyinna, Jinshan He, Jiaxing Miao, Nargis Shaheen, Boyu Xia, Cankun Wang, Qin Ma, Matthew C Bernier, Bryan A Whitson, Nuo Sun, Jing Zhao, Yutong Zhao

**Affiliations:** ^1^ Department of Physiology and Cell Biology The Davis Heart and Lung Research Institute The Ohio State University Columbus OH USA; ^2^ Department of Pathology The Ohio State University Columbus OH USA; ^3^ Department of Biomedical Informatics The Ohio State University Columbus OH USA; ^4^ Campus Chemical Instrument Center The Ohio State University Columbus OH USA; ^5^ Department of Surgery The Ohio State University Columbus OH USA; ^6^ Department of Internal Medicine The Ohio State University Columbus OH USA

**Keywords:** deubiquitinase, DNA methylation, endothelial barrier integrity, miRNA, SAM cycle, USP30

## Abstract

Microvascular dysfunction is a key contributor to the development of acute inflammatory diseases, characterized by heightened vascular hyperpermeability and leukocyte infiltration into interstitial tissues. Despite substantial research efforts, the precise mechanisms remain partially elucidated. Here, it is identified that USP30 is a critical regulator of lung microvascular inflammation and endothelial cell (EC) barrier integrity. Lipopolysaccharide (LPS) induces deubiquitinase activity of USP30. It is demonstrated that USP30 activation exacerbates EC dysfunction. Inhibiting USP30 leads to a 50% attenuation of inflammatory responses in ECs. In vivo, EC‐specific USP30‐deficient mice exhibit reduced microvascular dysfunction in models of endotoxin‐induced and ischemia‐reperfusion lung injury. Inhibition of USP30 preserves EC function via a mitophagy‐independent mechanism involving the S‐adenosylmethionine (SAM) cycle, DNA methylation, and miR‐30a‐5p expression. Mechanistically, USP30 depletion destabilizes and reduces methionine adenosyltransferase 2A (MAT2A) by deubiquitination, which in turn lowers SAM levels by ≈40%, and decreases global DNA methylation by roughly 35%, thereby resulting in a fourfold upregulation of miR‐30a‐5p. Elevated miR‐30a‐5p suppresses MDM2 and NFAT5 expression, contributing to the maintenance of EC function. These findings highlight that targeting USP30 may represent a potential therapeutic strategy warranting further preclinical and clinical exploration in acute lung injury.

## Graphical abstract

USP30 deubiquitinates and stabilizes MAT2A, thereby regulating DNA methylation and miRNA expression, which ultimately leads to disruption of lung endothelial barrier and inflammation.

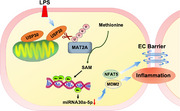

## 1 Introduction

Microvascular inflammation and barrier disruption play critical roles in the pathogenesis of acute inflammatory diseases,^[^ ^1^, ^2^, ^3^ ^]^ including *Pseudomonas aeruginosa*‐ or SARS‐CoV‐2‐induced acute respiratory distress syndrome (ARDS) and sepsis. In response to inflammatory stimuli, such as bacterial endotoxin, endothelial cells (ECs) increase the expression of chemokines and adhesion molecules, including interleukin‐8 (IL‐8), vascular cell adhesion molecule 1 (VCAM1), and intercellular adhesion molecule 1 (ICAM1), ultimately leading to neutrophil recruitment and transmigration, disruption of the endothelial barrier, and subsequent lung tissue injury.^[^ ^4^, ^5^, ^6^ ^]^ Therefore, preserving EC function represents a promising therapeutic strategy for managing acute inflammatory conditions.

De‐ubiquitinating enzymes (DUBs) regulate protein stability and activity by removing mono‐ubiquitin or polyubiquitin chains from ubiquitinated proteins.^[^ ^7^, ^8^, ^9^ ^]^ USP30, a DUB localized on the mitochondrial outer membrane,^[^ ^10^, ^11^ ^]^ is known for its canonical role in counteracting Parkin‐ and Pink1‐mediated mitochondrial mitophagy under cellular stress.^[^ ^10^, ^12^ ^]^ Mitophagy is an autophagic process that removes damaged mitochondria or dysfunctional mitochondria to maintain cellular homeostasis. Mitophagy plays divergent roles in EC function. Inhibition of mitophagy through elevated Parkin levels exacerbates hypertension‐induced dysfunction of the cerebrovascular endothelial barrier.^[^ ^13^ ^]^ In contrast, other studies have demonstrated that elevated mitophagy can promote ferroptosis and compromise endothelial barrier integrity.^[^ ^14^, ^15^ ^]^ Inhibition of USP30 has shown therapeutic potential in mitochondrial disorders, particularly in neurodegenerative diseases and cancers.^[^ ^12^, ^16^, ^17^, ^18^, ^19^ ^]^ However, its role in endothelial inflammation and barrier function remains unexplored.

S‐adenosylmethionine (SAM), a key methyl donor in DNA methylation, is synthesized from methionine by methionine adenosyltransferases (MAT1A and MAT2A).^[^ ^20^, ^21^ ^]^ DNA methylation is a major epigenetic mechanism that suppresses gene expression, including that of microRNAs (miRNAs).^[^ ^22^, ^23^, ^24^, ^25^, ^26^ ^]^ Elevated SAM levels enhance DNA methylation.^[^ ^20^, ^21^ ^]^ MiR‐30a‐5p has been implicated in regulating endothelial‐to‐mesenchymal transition, cell proliferation, and EC senescence,^[^ ^27^, ^28^, ^29^ ^]^ and it protects ECs from hypoxia‐induced apoptosis.^[^ ^28^, ^30^ ^]^ Furthermore, exogenous miR‐30a‐5p demonstrates an anti‐inflammatory property by attenuating LPS‐induced inflammatory responses in both glial cells and A549 cells.^[^ ^31^, ^32^ ^]^ Its expression is known to be downregulated by promoter methylation in glomerular podocytes.^[^ ^33^, ^34^ ^]^ However, the role of miR‐30a‐5p in EC inflammation and barrier function, as well as its regulatory mechanisms in ECs, remains unclear.

In this study, we investigate the role of USP30 in maintaining microvascular integrity. We demonstrate that USP30 depletion in ECs mitigates EC dysfunction under inflammatory conditions and in three experimental models of lung injury. Our findings uncover a mitophagy‐independent pathway involving USP30, the SAM cycle, DNA methylation, and miRNA regulation, suggesting that USP30 may serve as a novel therapeutic target for treating microvascular dysfunction in acute inflammatory diseases.

## 2 Results

### 2.1 LPS Increases USP30 Activity

**Figure 1:**
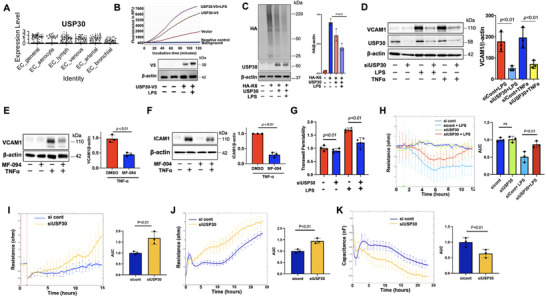
Inhibiting USP30 diminishes HLMVEC inflammation and permeability. A) Re‐analysis of lung single‐cell RNA‐seq data shows USP30 expression in human lung EC subtypes. B) Immunoprecipitated USP30‐V5 from USP30‐V5 plasmids‐transfected HEK293/TLR4 cells was incubated with Ubi‐AMC. Released AMC fluorescence was monitored using a plate reader with excitation and emission wavelengths at 360 and 460 nm, respectively. Overexpression of USP30‐V5 was confirmed with immunoblotting with V5 antibody. Images shown were representative graph images and immunoblots from three independent experiments. C) Immunoblotting analysis of poly‐HA‐K6‐ubiquitination (smeared bands) in K6‐ubiquitin‐HA and USP30 plasmids‐transfected HEK293/TLR4 cells with or without LPS (0.1 µg mL^−1^, 3 h) treatment. Data shown as mean ± SEM. Images shown were representative immunoblots from three independent experiments. D) VCAM1 levels were examined and quantified in USP30 siRNA‐transfected HLMVECs after treatment with LPS (0.1 µg mL^−1^) or TNFα (5 ng mL^−1^) for 6 h. Data shown as mean ± SEM. Images shown were representative immunoblots from three independent experiments. E,F) VCAM1 and ICAM1 levels were examined and quantified in MF‐094 (20 µM, 48 h)‐pretreated HLMVECs after treatment with TNFα (5 ng mL^−1^) for 6 h. Data shown as mean ± SEM. Images shown were representative immunoblots from three independent experiments. G) HLMVECs were treated with LPS (0.1 µg mL^−1^, 6 h). FITC‐dextran (70 kDa) leakage assay using trans‐well inserts with 0.4 µm pore membrane. n = 4. Data shown as mean ± SEM. H) HLMVECs (sicont or siUSP30‐transfected) were grown on ECIS chambers to confluence, followed by the addition of LPS (0.1 µg mL^−1^). TEER was measured by the ECIS system. Reduction of TEER indicates an increase in permeability. I) HLMVECs (sicont or siUSP30‐transfected) were grown on an ECIS chamber to confluence. Cells were electrically injured, and then TEER was measured. J,K) Suspended HLMVECs (sicont or siUSP30‐transfected) were loaded on ECIS chambers. TEER and capacitance were immediately measured for resistance (I) and capacitance (J). Data shown as mean ± SEM, n = 3.

USP30, a mitochondrial outer membrane deubiquitinase,^[^ ^10^, ^11^ ^]^ has been implicated in neurodegeneration and cancer,^[^ ^16^, ^35^, ^36^, ^37^ ^]^ but its role in endothelial function remains unexplored. Re‐analysis of lung single‐cell RNA‐seq data (www.LungEndothelialCellAtlas.com) revealed that USP30 is ubiquitously expressed across all EC sub‐types, including gCap (general capillary) and aCap (aeroctye) ECs (**Figure** **1**A). To assess its relevance in acute inflammation, we examined USP30 activity following lipopolysaccharide (LPS) stimulation. In TLR4/CD14‐overexpressing HEK293 cells transfected with V5‐tagged USP30, LPS treatment significantly increased USP30 deubiquitinase activity (Figure 1B). Consistent with its known specificity for Lys (K)6 ‐linked ubiquitin chains.^[^ ^38^, ^39^ ^]^ USP30 overexpression reduced K6‐linked polyubiquitination, an effect that was elevated by LPS treatment (Figure 1C), indicating that inflammatory stimuli enhance USP30 deubiquitinase activity.

### 2.2 USP30 Inhibition Attenuates Endothelial Inflammation and Barrier Dysfunction

Given the central role of EC inflammation and barrier dysfunction in acute inflammatory diseases,^[^ ^1^, ^2^, ^3^ ^]^ we investigated whether USP30 modulates these processes. Upregulation of VCAM1 and ICAM1 is the feature of inflamed ECs.^[^ ^40^ ^]^ Both LPS and TNFα treatment upregulated VCAM1 and ICAM1 expression in human lung microvascular cells (HLMVECs), while USP30 downregulation or pharmacological inhibition with MF‐094^[^ ^41^ ^]^ significantly suppressed this upregulation (Figure 1D–F), suggesting that USP30 is activated under inflammatory conditions, and it exhibits a pro‐inflammatory effect in ECs. Preservation of microvascular barrier integrity is important for maintaining tissue homeostasis. Next, we determined the effect of USP30 knockdown on LPS‐induced EC hyperpermeability by trans‐well permeability assay and electric cell‐substrate impedance sensing (ECIS) system. LPS increased FITC‐labeled dextran leakage (Figure 1G) and lowered trans‐endothelial resistance (TEER) (Figure 1H), indicating that LPS induced EC hyperpermeability. The detrimental effects of LPS were attenuated in USP30‐downregulated cells in both assays (Figure 1G,H), suggesting that inhibiting USP30 protects against LPS‐induced EC hyperpermeability. Furthermore, USP30 knockdown increased HLMVEC migration (Figure 1I), proliferation (Figure 1J), and adhesion (Figure 1K). Taken together, these data reveal that inhibiting USP30 preserves EC function under inflammatory stress.

### 2.3 EC‐Specific USP30 Knockout Mice are Protected From Acute Lung Injury

**Figure 2:**
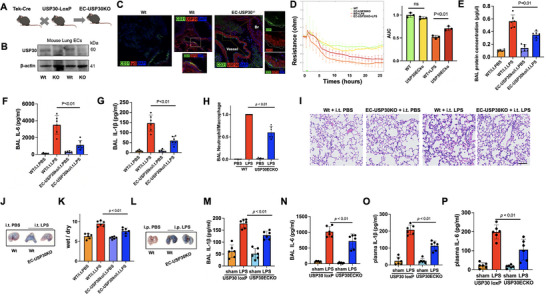
Depletion of USP30 in ECs ameliorates endotoxin‐induced acute lung injury. A) Scheme shows the targeting strategy for generating the EC‐USP30KO mice. B) Immunoblotting analysis of USP30 from isolated lung ECs from Wt and EC‐USP30KO mice. C) USP30 expression was deleted in CD31‐positive cells in EC‐USP30KO mouse lungs, compared to wild‐type (Wt) mice. IgG was used as a negative control. Br, Bronchus. D) Isolated lung ECs (from Wt and EC‐USP30KO mice) were cultured on ECIS chambers to confluence. TEER was measured for cell permeability. Data shown as mean ± SEM, n = 3. E–H) Mice were challenged with (i.t.) LPS (5 mg kg^−1^ body weight) for 24 h. Protein levels (E), IL‐6 (F), IL‐1β (G), and neutrophil influx (H) in BAL were measured. Data shown as mean ± SEM, n = 6. I) H&E staining of lung tissues. Scale bar, 50 µm. J) Evans Blue in vivo leakage assay after (i.t.) LPS. K) Lung wet/dry ratio was measured. L) Mice were challenged with (i.p.) LPS (2 mg kg^−1^ body weight) for 24 h. Evans Blue in vivo leakage assay after (i.p.) LPS. Data shown as mean ± SEM, n = 6. M,N) IL‐1β and IL‐6 in BAL were measured by ELISA. Data shown as mean ± SEM, n = 6. O,P) IL‐1β and IL‐6 in plasma were measured by ELISA. Data shown as mean ± SEM, n = 6.

**Figure 3:**
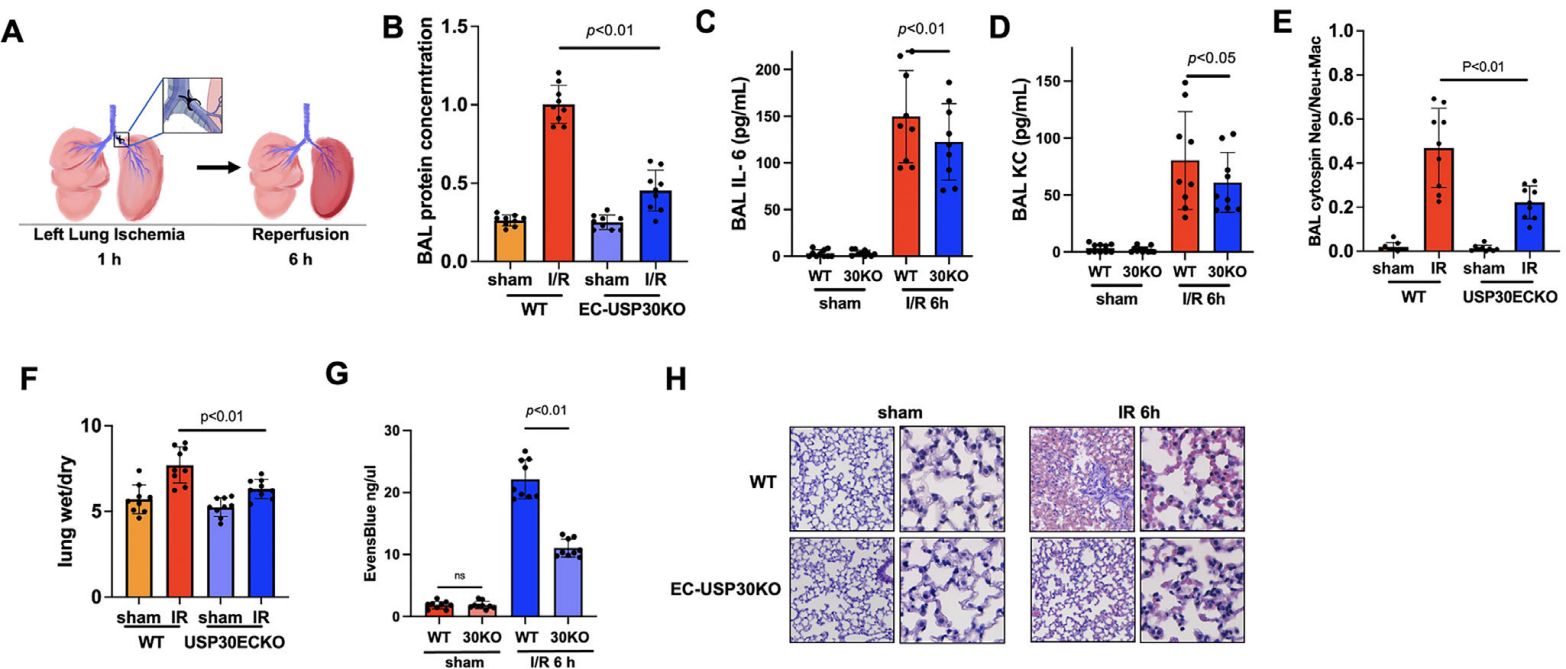
Depletion of USP30 in ECs ameliorates LIRI. A) The scheme shows the surgical method. B‐E) Protein levels (B), IL‐6 (C), KC (D), and neutrophil influx (E) in BAL were measured. F) Left lung wet/dry ratio was measured. Data shown as mean ± SEM, n = 9. G) Quantification of Evans Blue in vivo leakage assay. Data shown as mean ± SEM, n = 9. H) H&E staining of lung tissues. Scale bar, 50 µm.

To validate these findings in vivo, we generated EC‐specific USP30‐deficient mice (EC‐USP30KO) (**Figure** **2**A). Genotyping was confirmed by PCR (Figure S1A, Supporting Information). Successful deletion was confirmed by genotyping PCR, immunoblotting, and immunofluorescence (Figure 2A–C). USP30 mRNA levels were depleted in isolated ECs, but not in isolated fibroblasts from lungs of EC‐USP30KO mice (Figure S1B, Supporting Information). ECs isolated from EC‐USP30KO mice exhibited resistance to LPS‐induced EC barrier disruption (Figure 2D). We utilized three murine models of acute lung injury to investigate whether depletion of USP30 in ECs diminishes microvascular dysfunction and severity of acute lung injury. Intratracheal (i.t.) LPS mimics bacterial infection‐induced ALI by directly targeting the lungs; intraperitoneal (i.p.) LPS serves as a well‐established model for mimicking sepsis‐induced ALI; lung ischemia‐reperfusion injury (LIRI) effectively mimics the ALI associated with lung transplantation, extracorporeal memrane oxygenation (ECMO), pulmonary embolism, and cardiac arrest. (i.t.) instillation of LPS (from *E. coli* serotype O127:B8, 2 mg (1 000 000 EU) kg^−1^, 24 h) increased bronchoalveolar (BAL) protein levels (an index of vascular leakage, Figure 2E), IL‐6 (Figure 2F), IL‐1β (Figure 2G), and neutrophil influx (Figure 2H) in wild type mice, but all these effects were diminished in EC‐USP30KO mice. Furthermore, H&E staining revealed that EC‐USP30KO mice demonstrate reduced inflammatory cell infiltration compared to wild‐type mice (Figure 2I). Lung vascular permeability was assessed by Evans blue in vivo leakage assay and lung wet/dry weight ratio. EC‐USP30KO mice reduced LPS challenge‐induced Evans blue accumulation (Figure 2J) in lungs and lung wet/dry ratio (Figure 2K). In addition to the direct lung injury model, a systemic endotoxin (i.p. LPS)‐induced lung injury model (Figure 2L–P) and LIRI (,48Figure 3) were used. EC‐USP30 depletion reduced Evans blue leakage into lung tissues (Figure 2L), BAL IL‐1β (Figure 1M), BAL IL‐6 (Figure 2N), plasma IL‐1β (Figure 2O), and plasma IL‐6 (Figure 2P) after (i.p.) injection of LPS (5 mg (2 500 000 EU) kg^−1^, 24 h). Furthermore, lung I/R (**Figure** **3**A) increased left lung BAL protein concentration (Figure 3B), IL‐6 (Figure 3C), KC (Figure 3D), neutrophil influx (Figure 3E), wet/dry weight ratio (Figure 3F), and Evans blue leakage into interstitial tissues (Figure 3G) in wild type mice, while depletion of EC‐USP30 ameliorated lung I/R‐induced lung inflammation and edema. Left lung H&E staining suggests EC‐USP30 depletion reduced the severity of lung injury (Figure 3H). The consistent in vivo and in vitro data suggest that inhibiting USP30 preserves EC function and alleviates lung injury.

### 2.4 USP30 Knockdown Upregulates miR‐30a‐5p

**Figure 4:**
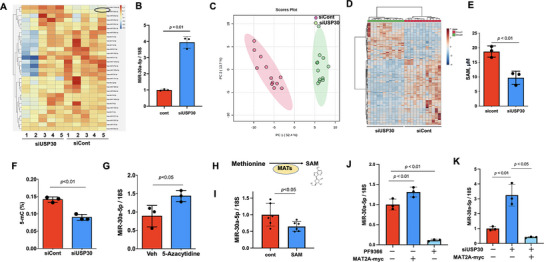
Depletion of USP30 increases miR‐30a‐5p expression and decreases SAM and DNA methylation in ECs. A) HLMVECs (n = 5) were treated with siCont or siUSP30 for 3 days, and miRNAs were extracted for miRNA‐seq analysis. Shown is a heat map of miRNA expression. MiR‐30a‐5p is highlighted by the black circle. B) Real‐time PCR analysis of miR‐30a‐5p levels in USP30 siRNA‐transfected HLMVECs. Data shown as mean ± SEM, n = 3. C) Score plot of samples from siCont group and the siUSP30 group using the PLS‐DA model. D) HLMVECs (n = 12) were treated with siCont or siUSP30 for 3 days, and then subjected to metabolic analysis. Clustering result shown as a heatmap. Distance measure using Euclidean, and clustering algorithm using ward's method. E) ELISA analysis of SAM levels in USP30 siRNA‐transfected HLMVECs. Data shown as mean ± SEM, n = 3. F) ELISA analysis of global 5‐methylcytosine (5‐mc) in DNA samples from USP30 siRNA‐transfected HLMVECs. Data shown as mean ± SEM, n = 3. G) Real‐time PCR analysis of miR‐30a‐5p levels in 5‐azacytidine (2 µM, 24 h)‐treated HLMVECs. Data shown as mean ± SEM, n = 3. H) The Scheme shows SAM production by MATs. I) Real‐time PCR analysis of miR‐30a‐5p levels in 5‐azacytidine (0.1 mM, 10 h)‐treated HLMVECs. Data shown as mean ± SEM, n = 6. J) Real‐time PCR analysis of miR‐30a‐5p levels in PF9366 (40 µM, 24 h) or MAT2A‐myc‐transfected HLMVECs. Data shown as mean ± SEM, n = 3. K) Real‐time PCR analysis of miR‐30a‐5p levels in USP30 siRNA‐ or USP30 siRNA+MAT2A‐myc‐transfected HLMVECs. Data shown as mean ± SEM, n = 3.

To further explore the mechanisms by which USP30 inhibition reduces EC inflammation and preserves EC barrier integrity, we examined pathways related to EC inflammation and barrier function, including phosphorylation of NF‐κB and p38 MAPK. None were observed to be altered in USP30 knockdown cells compared to control cells in response to LPS or TNFα (Figure S2, Supporting Information). Accumulating evidence shows that miRNAs regulate vascular development, growth, inflammatory responses, and barrier integrity,^[^ ^42^, ^43^, ^44^ ^]^ but no previous study has demonstrated a role of USP30 in the regulation of miRNA expression. We performed an unbiased miRNA‐seq and found that knockdown of USP30 regulated the expression of multiple miRNAs. MiR‐30a‐5p was one of the top upregulated miRNAs in USP30‐knockdown HLMVECs (**Figure** **4**A), and that was confirmed by real‐time PCR (Figure 4B). To further investigate the mechanisms by which inhibiting USP30 increases miR‐30a‐5p, we investigated the effect of USP30 knockdown on mitochondrial function. Mitochondrial superoxide production (Figure S3A, Supporting Information) and mitophagy markers (Figure S3B, Supporting Information) were not significantly altered by USP30 knockdown. We have shown that inhibiting USP30 increased ATP production in HeLa cells.^[^ ^19^ ^]^ Furthermore, we found that knockdown of USP30 also increased mitochondrial respiration and ATP production in HLMVECs (Figure S3C,D, Supporting Information), supporting that endothelial USP30 regulates mitochondrial function, but not mitochondria‐derived ROS production and mitophagy at least during the acute phase of EC inflammation and barrier disruption. Increases in ATP after USP30 deletion do not seem to be a major mechanism for miR‐30a‐5p upregulation. Thus, we need further investigation of non‐canonical mechanisms by which USP30 inhibition increases miR‐30a‐5p levels.

### 2.5 USP30 Regulates miR‐30a‐5p via SAM Metabolism and DNA Methylation

Cell metabolism plays a role in epigenetic regulation of transcription processing. The role of USP30 in cell metabolism remains unknown. We analyzed cell metabolism changes in USP30 siRNA‐transfected HLMVECs by LC‐MS. Partial least‐squares discriminant analysis (PLS‐DA) shows there was an obvious separation among siControl and siUSP30 groups (Figure 4C). Dramatic changes of a variety of cell metabolites were observed (Figure 4D), including the pentose phosphate pathway, purine metabolism, alanine, aspartate, and glutamate metabolism, etc. SAM is the main cellular methyl donor of DNA methylation. We found that SAM levels were decreased in USP30 siRNA‐transfected HLMVECs (Figure 4E). This was accompanied by decreased global DNA methylation (Figure 4F). Further, consistent with previous reports,^[^ ^33^, ^34^ ^]^ inhibition of DNA methylation by 5‐Azacytidine increased miR‐30a‐5p expression (Figure 4G). Methionine adenosyltransferases (MATs) are responsible for the biosynthesis of SAM from methionine (Figure 4H). Supplementation of SAM decreased miR‐30a‐5p levels, indicating that DNA methylation suppresses miR‐30a‐5p expression (Figure 4I). Furthermore, inhibition of MAT2A by PF9366 increased miR‐30a‐5p; conversely, overexpression of MAT2A decreased miR‐30a‐5p (Figure 4J). Elevated miR‐30a‐5p in USP30‐deficient cells was attenuated by overexpression of MAT2A (Figure 4K). These results link USP30 to epigenetic regulation via the SAM cycle.

### 2.6 USP30 Deubiquitinates and Stabilizes MAT2A

**Figure 5:**
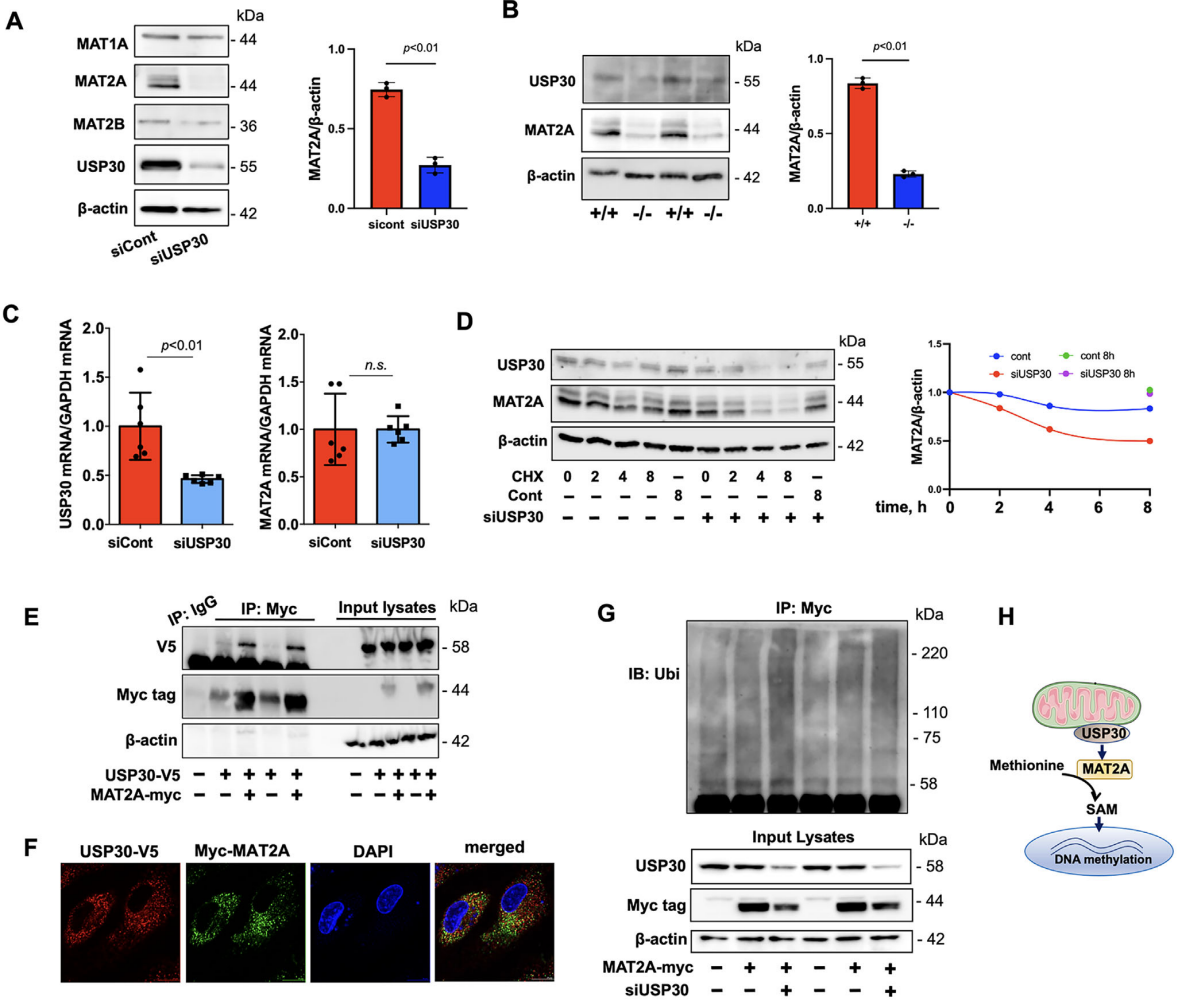
USP30 deubiquitinates and stabilizes MAT2A. A) Immunoblotting analysis of MAT isoforms in USP30 siRNA‐transfected HLMVECs. Data shown as mean ± SEM, n = 3. Images shown were representative immunoblots from three independent experiments. B) Immunoblotting analysis of MAT2A in isolated lung ECs from wild‐type and EC‐USP30KO mice. Data shown as mean ± SEM. C) Real‐time PCR analysis of *USP30* and *MAT2A* mRNA in USP30 siRNA‐transfected HLMVECs. Data shown as mean ± SEM, n = 6. D) Immunoblotting analysis of MAT2A in CHX (20 µM)‐treated HLMVECs (sicont or siUSP30‐transfected). E) USP30‐V5 and MAT2A‐myc‐overexprssed HEK293/TLR4 cells were subjected to immunoprecipitation with Myc tag antibody, followed by V5 and Myc tag immunoblotting (2 sets). F) Co‐immunofluorescence staining of USP30‐V5 and Myc‐MAT2A in HLMEVCs. Yellow dots in the merged image indicate co‐localization of the two proteins. Scale bar, 10 µm. G) HEK293/TLR4 cells were transfected with USP30 siRNA and MAT2A‐myc plasmid. Denatured cell lysates were subjected to immunoprecipitation with Myc tag antibody, followed by ubiquitin immunoblotting (2 sets). H) Scheme shows that USP30 stabilizes MAT2A and increases SAM production and DNA methylation.

We found that among MAT's isoforms, MAT2A was reduced in USP30 siRNA‐transfected HLMVECs (**Figure** **5**A; Figure S4, Supporting Information) and isolated lung endothelial cells from EC‐USP30KO mice compared to USP30‐LoxP mice (Figure 5B). To investigate the molecular mechanisms by which inhibiting USP30 reduces MAT2A levels, we examined the MAT2A mRNA levels and found that downregulation of USP30 did not alter MAT2A gene transcript levels (Figure 5C), suggesting that the reduction of MAT2A by inhibiting USP30 is possibly due to elevated protein degradation. To date, no study has investigated MAT2A degradation. We found that MAT2A degradation was inhibited by the lysosome inhibitor (leupeptin), not the proteasome inhibitor (MG‐132). Co‐immunofluorescence staining demonstrates the co‐localization of MAT2A with lysosomes, indicating that MAT2A degradation is mediated by the lysosome (Figure S5, Supporting Information). Mechanistically, knockdown of USP30 shortened MAT2A's half‐life (Figure 5D). Co‐immunoprecipitation and co‐immunofluorescence staining verified that MAT2A is associated with USP30 (Figure 5E,F). Further, downregulation of USP30 increased MAT2A polyubiquitination (Figure 5G), establishing MAT2A as a novel USP30 substrate (Figure 5H).

### 2.7 USP30‐miR‐30a‐5p Axis Regulates MDM2 and NFAT5

**Figure 6:**
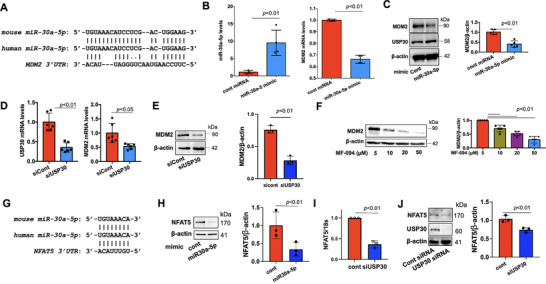
MiR‐30a‐5p targets and suppresses *MDM2* and *NFAT5* gene expression. A) Predicted targeting site of human and mouse miR‐30a‐5p on *MDM2* 3′‐UTR. B) Real‐time PCR analysis of miR‐30a‐5p levels and *MDM2* mRNA levels in miR‐30a‐5p mimic‐transfected HLMVECs. Data shown as mean ± SEM, n = 4. C) MDM2 immunoblotting analysis in miR‐30a‐5p mimic‐transfected HLMVECs. Data shown as mean ± SEM, n = 5. D, E) Real‐time PCR (D) and immunoblotting (E) analysis of MDM2 in USP30 siRNA‐transfected HLMVECs. Data shown as mean ± SEM, n = 3‐6. F) MDM2 immunoblotting analysis in MF‐094 (48 h)‐treated HLMVECs. Data shown as mean ± SEM, n = 5. G) Predicted targeting site of human and mouse miR‐30a‐5p on *NFAT5* 3′‐UTR. H) Immunoblotting analysis of *NFAT5* in miR‐30a‐5p mimic‐transfected HLMVECs. Data shown as mean ± SEM, n = 3. I,J) Real‐time PCR (I) or immunoblotting (J) analysis of NFAT5 in miR‐30a‐5p mimic‐transfected HLMVECs. Data shown as mean ± SEM, n = 3. Immunoblots were representative images from three to five independent experiments.

MiR‐30a‐5p mimic overexpression ameliorated LPS‐induced inflammatory responses in A549 cells^[^ ^32^ ^]^; however, the role of miR‐30a‐5p in lung microvascular integrity has not been reported. MiRNA TARGETSCAN software analysis revealed that *MDM2* was a possible target of both mouse and human miR‐30a‐5p and was mapped to the 3′UTR (**Figure** **6**A). To verify whether miR30a‐5p regulates MDM2 mRNA expression, we analyzed MDM2 mRNA levels in miR‐30a‐5p mimic‐transfected HLMVECs and found that an increase in miR‐30a‐5p levels decreased MDM2 mRNA, as well as MDM2 protein levels, without altering USP30 levels (Figure 6B,C). As USP30 regulates miR‐30a‐5p, we hypothesized that USP30 modulates MDM2 expression. As shown in Figure 6D,E, downregulation of USP30 reduced MDM2 mRNA and protein expression. Inhibition of USP30 with MF‐094 decreased MDM2 protein levels in a dose‐dependent manner (Figure 6F). Furthermore, we found that miR‐30a‐5p downregulated the expression of transcriptional factor NFAT5 in HLMVECs (Figure 6G,H). Downregulation of USP30 also correspondingly reduced NFAT5 expression (Figure 6I,J), suggesting that we revealed a new regulatory cascade that links USP30/miR30a‐5p/MDM2 and NFAT5 in microvascular endothelial cells.

### 2.8 MiR‐30a‐5p/MDM2 and NFAT5 Axes Mediate Endothelial Inflammation and Barrier Integrity

**Figure 7:**
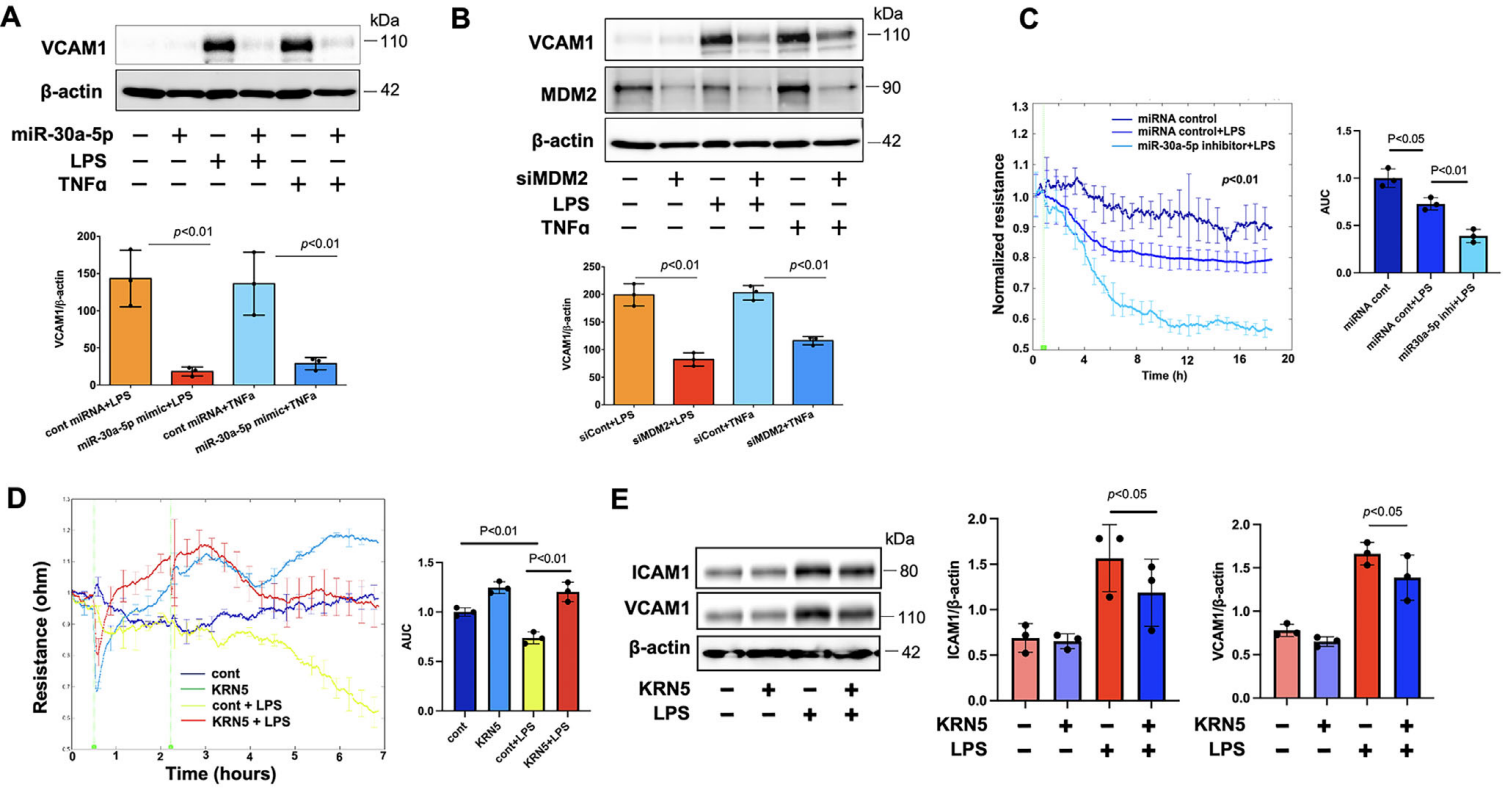
MiR30a‐5p/MDM2 or miR30a‐5p/NFAT5 attenuates EC inflammation and barrier dysfunction. A) Immunoblotting analysis of VCAM1 in miR‐30a‐5p mimic‐transfected HLMVECs after LPS or TNFα treatment for 6 h. Data shown as mean ± SEM, n = 3. B) Immunoblotting analysis of VCAM1 in MDM2 siRNA‐transfected HLMVECs after LPS or TNFα treatment for 6 h. Data shown as mean ± SEM, n = 3. C) HLMVECs were treated with miRNA control or miR‐30a‐5p inhibitor for 48 h, and then treated with LPS. EC permeability was measured by ECIS. Data shown as mean ± SEM, n = 3. D) HLMVECs were treated with DMSO control or KRN5 (5 µM) for 3 h, and then treated with LPS (0.1 µg mL^−1^). EC permeability was measured by ECIS. Data shown as mean ± SEM, n = 3. E) HLMVECs were treated with DMSO control or KRN5 (5 µM) for 3 h, and then treated with LPS (0.1 µg mL^−1^, 6 h). ICAM1 and VCAM1 levels were analyzed by immunoblotting. Data shown as mean ± SEM, n = 3. Images shown were representative immunoblots from three independent experiments.

As discovered above, USP30 regulates EC inflammation and barrier function, as well as modulates miR‐30a‐5p/MDM2 and NFAT5 expression. MDM2 and NFAT5 have been shown to promote inflammatory responses as co‐transcription factors for NF‐κB in the nuclei;^[^ ^45^, ^46^ ^]^ thus, we hypothesized that the miR‐30a‐5p/MDM2 and NFAT5 axes may regulate EC inflammation. HLMVECs were transfected with miR‐30a‐5p mimic or MDM2 siRNA, or treated with NFAT5 inhibitor (KRN5) followed by treatment with LPS or TNFα for 6 h. As shown in **Figure** **7**A,B, elevation of miR‐30a‐5p or downregulation of MDM2 attenuated VCAM1 expression in HLMVECs. Furthermore, miR‐30a‐5p inhibitor promoted LPS‐induced hyperpermeability in HLMVECs (Figure 7C), suggesting that the miR‐30a‐5p/MDM2 axis, the downstream pathway of USP30, regulates EC inflammation and barrier integrity. Inhibition of NFAT5 significantly protected against LPS‐induced EC hyperpermeability (Figure 7D). KRN5 slightly attenuated LPS‐induced ICAM1 and VCAM1 expression (Figure 7E), suggesting that the miR‐30a‐5p/NFAT5 axis majorly regulates microvascular EC permeability. Together, the data revealed a potential cascade: inhibiting USP30 ⇒ decreased the SAM cycle ⇒ increased miR‐30a‐5p ⇒ decreased MDM2 and NFAT5 levels ⇒ preserved EC function.

## 3 Discussion

Microvascular hyper‐inflammation and barrier disruption are central features of acute inflammatory diseases, leading to inflammatory cell infiltration, tissue edema, and eventually organ dysfunction. While our previous study demonstrated that USP30 inhibition induces mitophagy in cardiac myocytes,^[^ ^47^ ^]^ its role in the regulation of microvascular function remains unknown. In this study, we provide the first evidence that USP30 is activated in response to endotoxin exposure and its inhibition protects lung microvascular ECs from inflammatory stimuli and experimental lung injury. Mechanistically, USP30 knockdown promotes degradation of MAT2A, a key enzyme in methyl donor synthesis, leading to reduced DNA methylation and increased expression of miR‐30a‐5p. Furthermore, we show that miR‐30‐5p downregulates MDM2 and NFAT5, two pro‐inflammatory mediators that impair EC barrier function. These findings reveal a novel, mitophagy‐independent role of USP30 in regulating endothelial inflammation and permeability.

USP30 is a mitochondrial deubiquitinase that removes polyubiquitin chains from mitochondrial outer membrane proteins. Overexpression of USP30 reduces mitophagy and promotes apoptosis;^[^ ^10^, ^11^, ^12^, ^16^, ^17^, ^18^, ^19^ ^]^ however, the role of USP30 in endothelial cells and inflammatory lung diseases has not been reported. Here, we show that USP30 activity is increased in response to LPS. It has been shown that USP30 can be phosphorylated and activated by IKKβ,^[^ ^17^ ^]^ which is a key downstream molecule of LPS/TLR4 signaling. Activation of IKKβ regulates EC inflammatory responses.^[^ ^48^ ^]^ The role of IKKβ in LPS‐induced USP30 phosphorylation and activation needs to be investigated in ECs in future studies. Here, we focus on the role of USP30 in microvascular EC inflammation and monolayer permeability. Our data reveal that inhibiting USP30 preserves EC integrity by hindering EC inflammatory responses and hyper‐permeability. Using three distinct murine models of acute lung injury, we demonstrated that endothelial‐specific USP30 knockout significantly reduces vascular leakage, cytokine production, and lung inflammation. The EC‐USP30KO mice were generated by crossing with the Tek‐cre^1Ywa/J^ allele; a small number of circulating cells have been reported to be Cre positive in adult mice. Based on the data from the in vitro EC cell barrier assay and lung permeability index from in vivo disease models, we conclude that the protective effects of newly generated mice are mainly caused by EC‐specific USP30 knockout.

While most studies on USP30 focus on its role in mitophagy, our data suggest that its impact on EC function is mediated through a novel epigenetic mechanism. USP30 deficiency only moderately regulates mitochondrial respiration, reactive oxygen species (ROS) production, and changes in mitophagy markers. Instead, we observed a significant upregulation of miR‐30a‐5p in USP30‐deficient ECs. MiR30a‐5p has been shown to ameliorate LPS‐induced inflammatory responses in A549 cells and microglia.^[^ ^31^, ^32^ ^]^ Further, we identified MDM2 and NFAT5 as downstream targets of miR‐30a‐5p and demonstrated that their inhibition mitigates LPS‐induced EC inflammation and hyperpermeability. To our knowledge, this is the first study uncovering a role of NFAT5 in EC hyperpermeability. MiR‐30a‐5p has been reported to target multiple gene expression in a variety of cell types,^[^ ^32^, ^49^, ^50^ ^]^ and these targets may also contribute to the protective role of miR‐30a‐5p in microvascular ECs. Recent studies suggested a beneficial role of EC‐derived exosomal miR‐30a‐5p in the inhibition of lung tumor growth and migration.^[^ ^51^ ^]^ The role of systemic exosomal miR‐30a‐5p in lung inflammatory disease has not been reported.

We further uncovered a mechanistic link between USP30 and DNA methylation. Increased DNA methylation of the miR‐30a‐5p promoter is associated with miR‐30a‐5p suppression.^[^ ^33^, ^34^ ^]^ Here, consistent with the previous reports, we show that inhibition of DNA methylation increases miR‐30a‐5p expression in lung ECs. Further, we revealed an uncovered mechanism by which inhibiting USP30 promotes ubiquitination and degradation of MAT2A, resulting in a decrease in methyl donor levels and DNA methylation. This elicits a molecular pathway that links mitochondria and nuclear DNA methylation through the SAM cycle. MAT2A is a key enzyme of SAM production;^[^ ^20^, ^21^ ^]^ however, the molecular regulation of MAT2A stability remains unknown. Here, we show that MAT2A degradation is mediated by ubiquitination and that USP30 negatively regulates MAT2A ubiquitination. Further studies on MAT2A stability may focus on identifying an ubiquitin E3 ligase for targeting MAT2A. The USP30/MAT2A/DNA methylation axis is an unveiled non‐canonical function of USP30. Inhibiting MAT2A has been shown to reduce inflammatory responses across a range of inflammatory conditions.^[^ ^52^, ^53^ ^]^ Direct evidence for anti‐inflammatory effects of MAT2A in endothelial cells remains lacking. Our study demonstrates that USP30 depletion destabilizes MAT2A and attenuates inflammatory responses in lung endothelial cells, supporting a potential anti‐inflammatory role of MAT2A. Another reported non‐canonical function of USP30 is regulation of ATP citrate lyase (ACL), a cytosolic enzyme that plays a critical role in acetyl CoA production.^[^ ^17^ ^]^ The role of USP30/ACL in lung EC barrier integrity remains further investigated. Despite these interesting results on the link between DNA methylation and miR‐30a‐5p expression, this study does have some limitations and future directions. DNA methylation may directly contribute to gene expression of inflammatory proteins and proteins related to EC barrier function. In the future, investigation is needed to disclose more mechanisms of USP30 regulation of EC integrity by focusing on USP30/DNA methylation, USP30/histone acetylation, and USP30/metabolism pathways.

In conclusion, our study identifies USP30 as a novel regulator of endothelial inflammation and barrier function through a previously unrecognized epigenetic mechanism involving MATA2A degradation, DNA methylation, and miR‐30a‐5p expression. These findings not only expand our understanding of mitochondrial‐nuclear communication in vascular biology but also highlight that targeting USP30 may represent a potential therapeutic strategy warranting further preclinical and clinical exploration in acute inflammatory diseases. USP30 inhibitor MTX325 is currently being tested in a clinical trial for Parkinson's Research. Future studies may evaluate MTX325 in a clinical trial as a potential treatment for acute inflammatory diseases.

## 4 Experimental Section

### Cells and Reagents

Human lung microvascular endothelial cells (HLMVECs, from ATCC) were cultured with endothelial growth medium‐2 (EGM‐2) at 37 °C in a cell culture incubator with 5% CO_2_. HEK293/TLR4/MD2 cells (from InvivoGen) were cultured in DMEM medium containing 10% fetal bovine serum (FBS). Antibodies for ICAM1, VCAM1, V5 tag, HA tag, Myc tag, USP30, NDP52, OPTN, p62, GAPDH, p‐p38, p‐p65, IQGAP1, MAT1A, MAT2A, MAT2B, MDM2, NFAT5, and β‐actin were from Cell Signaling (Danvers, MA), ProteinTech, and Santa Cruz Biotechnology (Santa Cruz, CA). The detailed dilution rate was listed in Table 1. Lipopolysaccharide (LPS), KRN5, and MF‐094 were purchased from MilliporeSigma (St. Louis, MO). Human recombinant TNFα was purchased from R&D Systems (Minneapolis, MN). All materials used in the experiments are the highest grade commercially available.

**Table 1:** Antibody information.

Antibody	Source	Dilution	
USP30	Mouse / IgG1	1:1000	Santa Cruz, sc‐515235
MAT1A	Rabbit / IgG	1:1000	Invitrogen, PA5‐41998
MAT2A	Rabbit / IgG	1:1000	Proteintech, 55309‐1‐AP
MAT2B	Rabbit / IgG	1:1000	Proteintech, 15952‐1‐AP
V5 tag	Mouse / IgG1	1:1000	Santa Cruz, sc‐81594
Myc Tag	Mouse IgG2a	1:1000	Cell signaling, #2276
HA‐tag	Rabbit / IgG	1:1000	Proteintech, 51064‐2‐AP
NFAT5	Mouse / IgG2a	1:1000	Santa Cruz, sc‐398171
NDP52	Rabbit / IgG	1:1000	Cell signaling, #60732
OPTN	Rabbit / IgG	1:1000	Proteintech, 10837‐1‐AP
MDM2	Mouse / IgG1	1:1000	Santa Cruz, sc‐965
Ubiquitin	Rabbit / IgG	1:1000	Proteintech, 10201‐2‐AP
ICAM‐1	Rabbit / IgG	1:1000	Santa Cruz, sc‐7891
VCAM‐1	Mouse / IgG1	1:1000	Santa Cruz, sc‐13160
IQGAP1	Rabbit / IgG	1:1000	Santa Cruz, sc‐10792
β‐Actin	mouse	1:2000	Sigma, A5441
GAPDH	Mouse / IgG2	1:2000	Proteintech, 60004‐1‐Ig
SQSTM1/p62	Rabbit / IgG	1:1000	Cell signaling, #39749
Phospho‐p38 MAPK (Thr180/Tyr182)	Rabbit / IgG	1:1000	Cell signaling, #9211
Phospho‐NF‐κB p65 (Ser536)	Rabbit / IgG	1:1000	Cell signaling, #3031

### Plasmid and siRNA Transfection

Human USP30 cDNA (NM_032663.5) was inserted into pCDNA3.1/V5‐His‐Topo vector, pCDNA3.1/HA vector. Human Myc‐MAT2A (NM_005911) plasmid was from OriGene (Rockville, MD). USP30 siRNAs and control siRNAs were from MilliporeSigma (St. Louis, MO). Plasmids and siRNAs transfections in ECs were performed using LipoJet or PepMute siRNA transfection reagent (SignaGen Laboratories, Frederick, MD). Overexpression or downregulation of genes was confirmed by immunoblotting and real‐time PCR. All the USP30 siRNA#1‐6 decreased USP30 levels (Figure S4, Supporting Information). In this study, USP30 siRNA#1 for the downregulation of USP30 was used.

### Immunoblotting and Co‐Immunoprecipitation (co‐IP)

Cell lysates were prepared in lysis buffer (Cell Signaling, Danvers, MA) with sonication at 4 °C. Equal amounts of total protein (20 µg) were subjected to SDS‐PAGE gel, transferred to nitrocellulose, and then immunoreacted with the primary and the secondary antibodies, sequentially. Membranes were stripped after the first set of antibodies and then re‐probed with another antibodies. For co‐IP, 1 mg of protein was incubated with a primary antibody overnight at 4 °C, followed by incubation with protein A/G beads for an additional 2 h at room temperature. The beads were rinsed with PBS and lysis buffer 4 times. Proteins on the beads were eluted by boiling in 2x SDS sample buffer and then subjected to immunoblotting. Immunoblot imaging was conducted using Azura Biosystems Imaging Station C600. Intensities of immunoblots were analyzed and quantified with Image J and normalized to the intensities of internal controls.

### In Vitro Ubiquitination Assay

Cells were washed with PBS, and then cell lysates were prepared in 50–80 µL of 2% SDS lysis buffer with ubiquitin aldehyde and N‐ethylmaleimide (NEM), followed by boiling for 10 min. This procedure dissociates non‐covalent protein–protein interaction. The denatured cell lysates were then sonicated on ice, followed by 10 times dilution with TBS. Cell lysates were subjected to immunoprecipitation, followed by immunoblotting.

### Trans‐Well Permeability Assay

HLMVECs were cultured to confluence on 12 mm trans‐well inserts with 0.4 µm pore membranes (Corning, Corning, NY), treated with LPS (0.1 µg mL^−1^) for 5.5 h, then subsequently incubated with 70 kDa FITC‐Dextran (1 mg mL^−1^) in the inserts. After 30 min, media were collected from receiver trays and inserts, and fluorescence was measured using a microplate reader (excitation 485 nm, emission 535 nm).

### Electrical Cell‐Substrate Impedance Sensing (ECIS) System

Transendothelial electrical resistance (TEER) measurement was conducted using the ECIS System (Applied BioPhysics, Troy, NY). HLMVECs were cultured on gold electrodes (8W10E) to confluence, treated with LPS (0.1 µg mL^−1^). The changes in resistance were continuously monitored in real‐time at a frequency of 4000 Hz. Decreases in TEER indicate hyperpermeability. For wound‐healing/cell migration assays, confluent HLMVECs cultured on gold electrodes (8W1E) were subjected to a current of 64 kHz, 1400 µA, and 30 s duration. For the cell proliferation and adhesion assay, suspended HLMVECs were loaded on gold electrodes (8W10E), and TEER and capacitance were continuously monitored in real‐time at frequencies of 4000 Hz and 60 kHz. Increases in TEER indicate increases in cell migration, adhesion, and proliferation. Decreases in capacitance indicate increases in cell adhesion and spreading. TEER and capacity values obtained from individual microelectrodes were aggregated at specific time intervals and presented as mean ± standard error of the mean (SEM). The area under the curve (AUC) (n = 3) was measured by Image J software.

### USP30 Activity Assay

HEK293 cells transfected with empty vector or the USP30‐V5 plasmid were collected, and USP30‐V5 was immunoprecipitated with V5 antibody, followed by incubation with IgG/A agarose beads. The beads were resuspended in deubiquitination assay buffer (40 mM Tris (pH 7.1), 100 mM NaCl, and 5 mM DTT) and deubiquitination activity was continuously monitored by incubation with ubiquitin‐AMC (UBPBio, Inc., Aurora, CO, USA), a fluorogenic substrate. Deubiquitinase activity was determined using fluorescence spectrophotometry with an excitation wavelength of 360 nm and an emission wavelength of 460 nm.

### Untargeted Metabolomics Analysis and SAM Assay

Untargeted metabolomics analysis was performed at the Metabolomics Core Facility at the Ohio State University. Briefly, proteins in siRNA control‐ and siUSP30‐transfected HLMVECs were precipitated by incubation with ice‐cold methanol for 30 min, followed by centrifugation. The supernatant was then aliquoted into LC vials for subsequent analysis. Prior to MS detection, samples were separated on a Poroshell 120 SB‐C18 column (2 × 100 mm^2^, 2.7 µm particle size) with an Agilent 1290 Infinity UHPLC system. All analyses were performed on an Agilent 6545 quadrupole time‐of‐flight mass spectrometer in sensitivity mode and positive polarity with electrospray ionization (ESI). MS/MS data‐dependent analysis was performed in which the top 5 ions were selected within a 30 s exclusion window, and all data sets were collected in centroid mode. For feature selection, including database comparison and statistical processing, samples were analyzed with Progenesis QI 3.0. SAM ELISA assay was performed according to manufacturer's instructions (Cell BioLabs, Inc., San Diego, CA).

### MicroRNA Sequencing (MiRNA‐seq) Analysis

Total RNA in siRNA control‐ and siUSP30‐transfected HLMVECs (n = 5) was extracted and purified using the miRNeasy kit (Qiagen, Germantown, MD), and 1 µg of total RNA was used for small RNA library preparation. MiRNA‐seq was performed by the Genomics Core Facility at the Ohio State University. Briefly, all RNA was validated using an Agilent Bioanalyzer. Illumina adapters were ligated to the RNA molecule, and cDNA was created by a reverse transcriptase reaction. Subsequently, PCR was carried out with a universal primer and a primer containing 1 of 48 Illumina index sequences (Illumina TruSeq SmallRNA kit). Amplified products were loaded on the Applied Biosystems SOLiD next‐generation high‐throughput sequencing system for data acquisition. The quality of the samples and libraries was verified on the Agilent Bioanalyzer.

### Realtime PCR

Total RNA was isolated from HLMVECs using the NucleoSpin RNA Extraction Kit (Clontech, Mountain View, CA) according to the manufacturer's instructions. RNA was quantified by Nano‐drop. cDNA was prepared using the iScript cDNA Synthesis Kit (Bio‐Rad). Quantitative PCR was performed to assess *USP30*, *MAT2A*, *NFAT5, MDM2*, and miR‐30a‐5p expression using iQ SYBR Green Supermix and the iCycler Real‐Time PCR Detection System (Bio‐Rad). *USP30* primers: 5′‐GGGCGGCCGTCAGATATAAA‐3′ and 5′‐ TTTCTGTAATGGGACCCCAAA‐3′. *NFAT5* primers: GTTGCTGCTGGATTTGCCTC‐3′ and 5′‐GAGGAAGAGGAGGGAGCTGA‐3′. *MAT2A* primers: 5′‐TACGAGTAGAACGCTGTCCG‐3′ and CGTTCATGTTGGTGTCGGTG‐3′. *MDM2* primers: 5′‐TGCCAAGCTTCTCTGTGAA‐3′ and 5′‐CGATGATTCCTGCTGATTGA‐3′. *GAPDH* primers: 5′‐GGGTCCCAGCTTAGGTTCAT‐3′ and 5′‐TACGGCCAAATCCGTTCACA‐3′. *MiR‐30a‐5p* primers: 5′TAGTCGAGGATGTTTATAG‐3′ and 5′‐AACTTCAATACTTTACAAAATCG‐3′. Gene expression was normalized to *GAPDH* levels.

### Immunofluorescence Staining

Cells grown on 35 mm glass bottom culture dishes were fixed in 3.7% formaldehyde for 20 min, followed by permeabilization with 0.1% Triton X‐100 for 2 min. After washing with PBS, cells were incubated with primary antibodies, followed by fluorescence‐labeled secondary antibodies. The fluorescence imaging was performed using a Nikon confocal microscope.

### DNA Methylation Assay

DNA methylation in the cells was measured using the Global DNA Methylation Assay Kit (ab233486, Abcam). Genomic DNA was extracted from the cells. A total of 100 ng of the diluted sample DNA and 100 µL of binding solution were added to each well of the microplate. For controls, 2 µL of negative and positive control solutions were added to the individual wells. The plate was then sealed and incubated at 37 °C for 60 min. After incubation, the wells were washed three times with 150 µL of Washing Buffer. 50 µL of the 5‐mC Detection Complex Solution (prepared by mixing Wash Buffer and 5‐mC antibody solution) was added to each well, and the plate was sealed and incubated at room temperature for 50 min. The wells were washed five times with the Washing Buffer after removal of the 5‐mC Detection Complex Solution. Then, 100 µL of Developer Solution was added to each well, followed by adding 100 µL of Stop Solution. Absorbance was measured at 450 nm.

### EC‐Specific USP30 Deficient Mice and Endotoxin‐ and Lung Ischemia Reperfusion (I/R)‐Induced Experimental Lung Injury Models

EC‐specific USP30‐deficient mice (EC‐USP30KO) were generated by breeding endothelial cell‐specific Cre transgenic mice (Tek‐Cre^1Ywa^ from Jackson Laboratory) and USP30‐fl/fl mice. All mice were housed in the specific pathogen‐free animal care facility at the Ohio State University in accordance with institutional guidelines and guidelines of the US National Institutes of Health. All animal experiments were approved by the Ohio State University Animal Resources Centers (Protocol #2018A00000122‐R2). Age‐matched males and females of C57/BL6J, or USP30‐fl/fl, and EC‐USP30KO mice (8–10 weeks) were given intratracheal (i.t.) LPS [*E. coli* serotype O127:B8, 5 mg (2 500 000 EU) kg^−1^ body weight] for 24 h or intraperitoneal (i.p.) LPS [2 mg (1 000 000 EU) kg^−1^ body weight] for 24 h. For the lung I/R injury model, mouse left main pulmonary artery was clamped with a 1‐0 silk suture for 1 h, then the left lung was reperfused by unclamping the left pulmonary artery for an additional 6 h. Bronchoalveolar lavage (BAL) fluid was collected for protein assay, cytokine assay, cytospin, and neutrophil accounting. Lung tissues were fixed for hematoxylin and eosin (H&E) staining. Lung Evans blue staining was performed by intravenous (i.v.) injection of Evans blue (20 mg kg^−1^, Biomedical MP LLC, USA) in the mouse lateral tail vein, followed by lung collection and imaging. To determine the lung wet‐to‐dry weight (W/D) ratio, left lung tissues were initially weighed upon removal (wet weight) and subsequently subjected to a 60 °C oven for 72 h. Then, the lung was reweighed (dry weight), and the ratio of the W/D weight was calculated to assess lung tissue edema. Randomization and blinded analysis for data analysis were performed.

### Statistical Analysis

Statistical analysis was carried out by one‐way or two‐way ANOVA, followed by Tukey's post hoc test or unpaired Student *t*‐test in comparing continuous measures. Data are expressed as mean ± SEM from at least three independent experiments. Sample numbers were indicated by dots. The value of *p*< 0.05 was considered statistically significant.

## Conflict of Interest

The authors declare no conflicts of financial interest.

## Author Contributions

B.B. and J.H. contributed equally to this work. B.B., J.H., J.M., B.X., N.S., C.W., Q.M., J.Z., and Y.Z. performed the experiments and data analysis. Y.Z. supervised the project. JY.Z. finalized the manuscript. B.W. guided the I/R model, provided suggestions on the studies, and edited the manuscript.

## Supporting information



Supporting Information

## Data Availability

The data that support the findings of this study are available from the corresponding author upon reasonable request.
