# Supplementary material for: Activation of USP30 Disrupts Endothelial Cell Function and Aggravates Acute Lung Injury Through Regulating the S‐Adenosylmethionine Cycle
Source: Adv Sci (Weinh). 2025 Oct 17;13(2):e12807. doi: 10.1002/advs.202512807 (PMC12786331; doi:10.1002/advs.202512807)
Supplement: Supplementary file 1 — Supporting Information [file ADVS-13-e12807-s001.pdf]

Supplemental data:

Activation of USP30 disrupts endothelial cell function and aggravates acute lung injury through regulating S-adenosylmethionine cycle

Baoyinna Baoyinna [1#], Jinshan He [1,2#], Jiaxing Miao [1], Nargis Shaheen [1], Boyu Xia [1], Cankun Wang [3], Qin Ma [3], Matthew C Bernier [4], Bryan A Whitson [5], Nuo Sun [1], Jing Zhao [1,6], Yutong Zhao [1,6*]

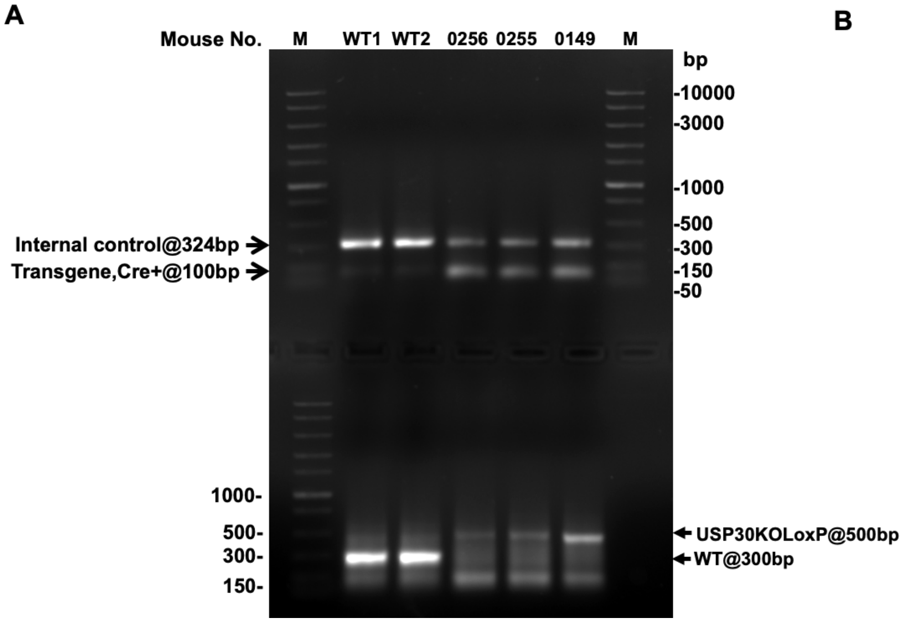

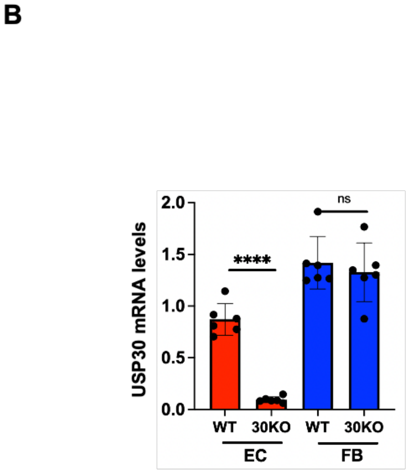

Supplemental figure 1. A. Genotyping of EC-USP30 mice by PCR. Upper PCR is with Cre primer; lower PCR is with LoxP primer. **B.** Realtime PCR analysis USP30 mRNA levels in isolated ECs and fibroblasts (FB) from wild type (WT) and EC-USP30KO mice. ****n<0.0001.

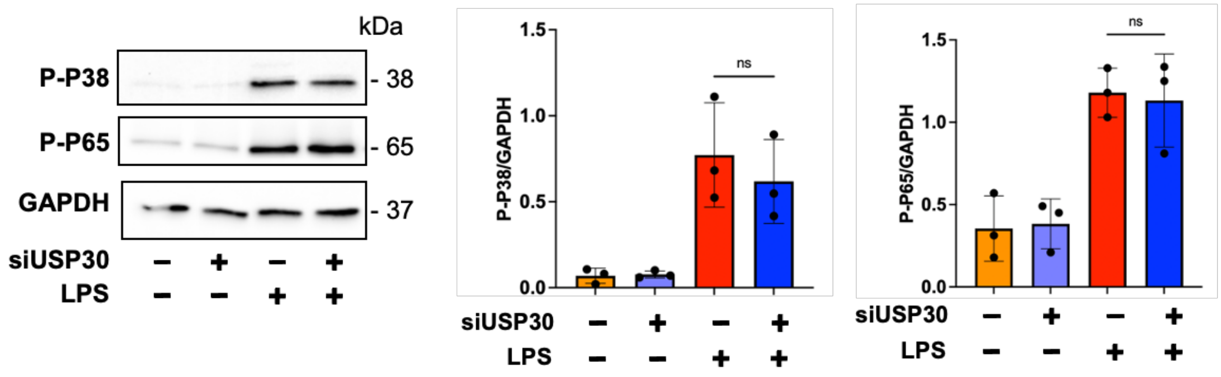

Supplemental figure 2. Immunoblotting analysis of p-P38 and p-P65 in LPS-treated HLMVECs (sicont or siUSP30-transfected).

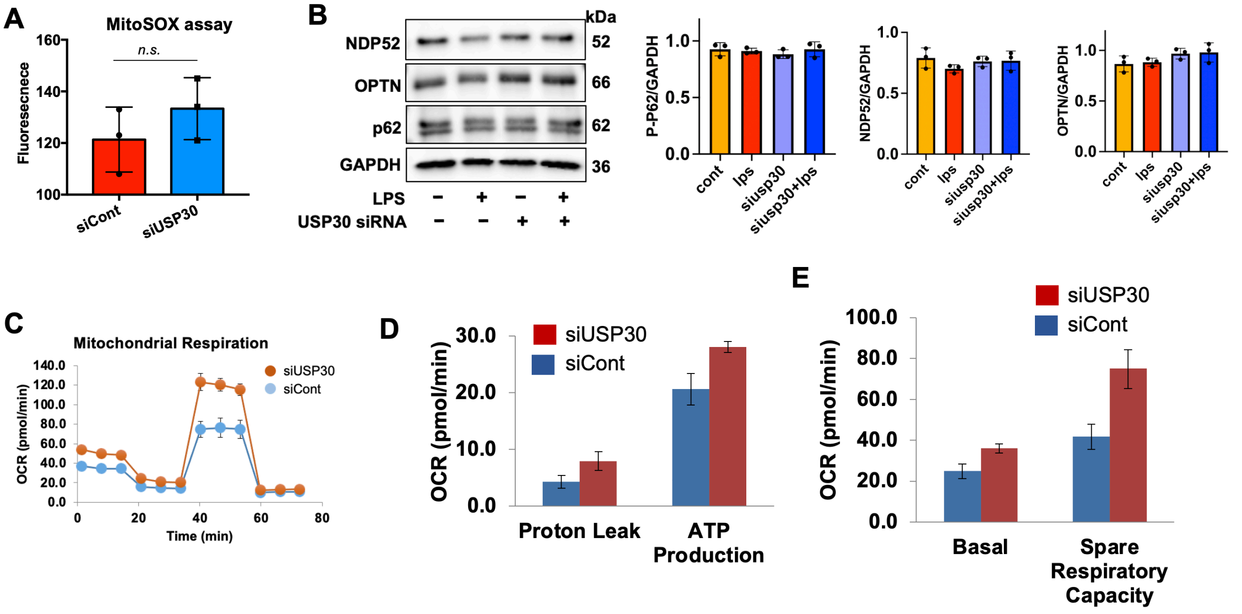

Supplemental figure 3. A. Quantification of MitoSOX staining in sicont and siUSP30-transfected HLMVECs. **B.** Immunoblotting analysis of NDP52, OPTN, and p62 in LPS-treated HLMVECs (sicont or USP30 siRNA-transfected). **C-E.** Seahorse assay results for OCRs of siUSP30-transfected HLMVECs. Data shown as mean ± SEM, n=3.

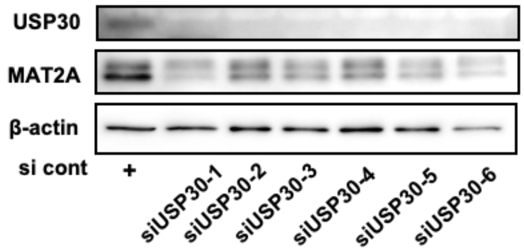

Supplemental figure 4. Immunoblotting analysis of USP30 and MAT2A in different USP30 siRNA (#1-6)-transfected HLMVECs.

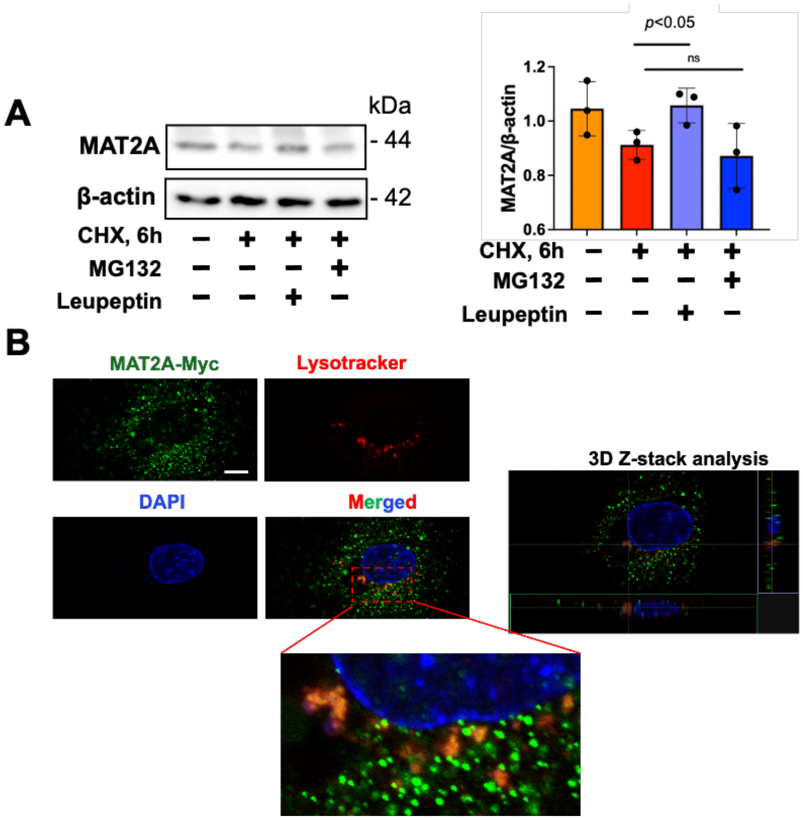

Supplemental figure 5. A. HLMVECs were treated with MG132 (20 µM) and leupeptin (100 µM) for 1 h, followed with CHX (20 µg/ml) for 6 h. MAT2A levels were analyzed by immunoblotting. **B.** HLMVECs grown on glass-bottom dishes were transfected with MAT2A-Myc plasmid for 48 h. Immunofluorescence staining was performed using antibody to Myc tag and lysotracker. Scale bar, 50 nm. 3D Z-stack analysis was performed using NIS-Elements software.
